# Geographic Variation in Antibiotic Consumption—Is It Due to Doctors’ Prescribing or Patients’ Consulting?

**DOI:** 10.3390/antibiotics7010026

**Published:** 2018-03-20

**Authors:** Marte Meyer Walle-Hansen, Sigurd Høye

**Affiliations:** 1 Bærum Hospital, Vestre Viken Hospital Trust, 3019 Drammen, Norway; 2 Antibiotic Centre for Primary Care, Department of General Practice, Institute of Health and Society, University of Oslo, 0315 Oslo, Norway; sigurd.hoye@medisin.uio.no

**Keywords:** antibiotic resistance, general practice, respiratory tract infections, drug consumption, pharmacoepidemiology

## Abstract

Antibiotic consumption varies greatly between Norwegian municipalities. We examine whether this variation is associated with inhabitants’ consultation rates or general practitioners’ (GP) prescription rates. Our study comprises consultations and antibiotic prescriptions for respiratory tract infections (RTIs) in general practice in all Norwegian municipalities with over 5000 inhabitants in 2014. Data was collected from The Norwegian Prescription Database, The Directorate of Health’s system for control and payment of health reimbursements registry and Norway Statistics. Consultation rates and prescription rates were categorised in age- and gender specific quintiles and the effect on antibiotic consumption was analysed using a Poisson regression model. We found that inhabitants with RTIs received 42% more prescriptions if they belonged to a municipality with high consultation rates compared to low consultation rates [incidence rate ratio (IRR) 1.42 (95% CI 1.41–1.44)] and 48% more prescriptions if they belonged to a municipality with high prescription rates versus low prescription rates [IRR 1.48 (95% KI 1.47–1.50)]. Our results demonstrate that inhabitants’ consultation rates and GPs’ prescription rates have about equal impact on the number of RTI antibiotics prescribed at municipality level. These findings highlight the importance of interventions targeting patients as well as doctors in efforts to reduce unnecessary antibiotic consumption.

## 1. Introduction

Antibiotics are an integral part of modern health care. The use of antibiotics, especially wide spectrum antibiotics, increases the risk of antimicrobial resistance both at national and local levels [1,2]. Scandinavian countries still have relatively low levels of antimicrobial resistance but this situation is threatened by an increasing relative use of broad spectrum antibiotics and import of resistant bacteria from abroad [3].

All inhabitants in Norway are entitled to a general practitioner (GP). GP offices, alongside accident and emergency units (A&Es), nursing homes, child health clinics and school health services make up most of Norwegian primary care. Most A&Es offer access 24 h a day year-round. Some GP offices offer appointments in the evenings on selected week days. Our study therefore includes consultations conducted after hours and on weekends.

Around 85% of all antibiotics in Norway measured in defined daily dose (DDD) are prescribed in primary care [3,4]. Around half of this amount is prescribed to treat respiratory tract infections (RTIs) [3], although the clinical benefit of antibiotic treatment is modest for most RTIs [5]. Several studies have explored ways to lower RTI antibiotic consumption in general practice. Interventions have especially targeted the antibiotic prescriber, aiming to reduce their prescription rate [6].

European countries show significant differences in antibiotic usage [7]. Variations in health care organisation and epidemiology cannot fully account for these differences [8]. Norwegian municipalities also show great variation in terms of antibiotics use per inhabitants and the number of prescriptions per municipality shows little variation over time [3,9]. Latitude and municipality population size has been shown to covariate with consumption but this effect may well be a surrogate for undisclosed variables such as different patient expectations, geographical distance between patient and health care provider and differing GP prescribing habits [10].

Retrospective analyses have demonstrated that time periods with lower rates of antibiotic consumption coincide with time periods where people frequent their doctor less often [11,12]. Many of the conditions for which patients visit their doctor are safely managed in the home with symptomatic treatment. This is especially relevant to RTIs. Antibiotic consumption is dependent both on the patients’ tendency to visit a doctor and the doctors’ tendency to prescribe antibiotics. The relative significance of these variables is not known.

To ensure a more sustainable use of antibiotics it is important to understand the mechanisms contributing to geographic differences in consumption. The aim of this study is to investigate to what degree geographic variations in antibiotic consumption for RTIs is associated with differences in inhabitants’ health seeking behaviour and GPs’ prescription behaviour.

## 2. Results

The data set comprises 3,364,585 inhabitants with a total of 1,037,278 primary care consultations for RTIs by the course of 2014. Study population details are provided in Table 1. There was a total of 738,646 RTI antibiotic prescriptions dispensed in the same year. RTI consultations, GPs’ prescription rates and RTI antibiotic prescriptions are summarised in Table 2. There was a weighted mean of 311 RTI consultations per 1000 inhabitants, making up 10.3% of all primary care consultations in 2014. GPs’ prescribed a weighted mean of 779 RTI prescriptions per 1000 RTI consultations and the rate increased with increasing age for both genders. There was a weighted mean of 220 RTI prescriptions dispensed per 1000 inhabitants the same year.

### Effect on the Total Use of Antibiotics

The findings from the Poisson regression analysis are summarised in Table 3. The outcome of interest was RTI antibiotic prescriptions per 1000 inhabitants. We found a lower risk of getting a prescription in males [incidence rate ratio (IRR) 0.78]. The risk of getting a prescription for treatment of an RTI was highest among preschool age and late retirement age.

**Figure 1:**
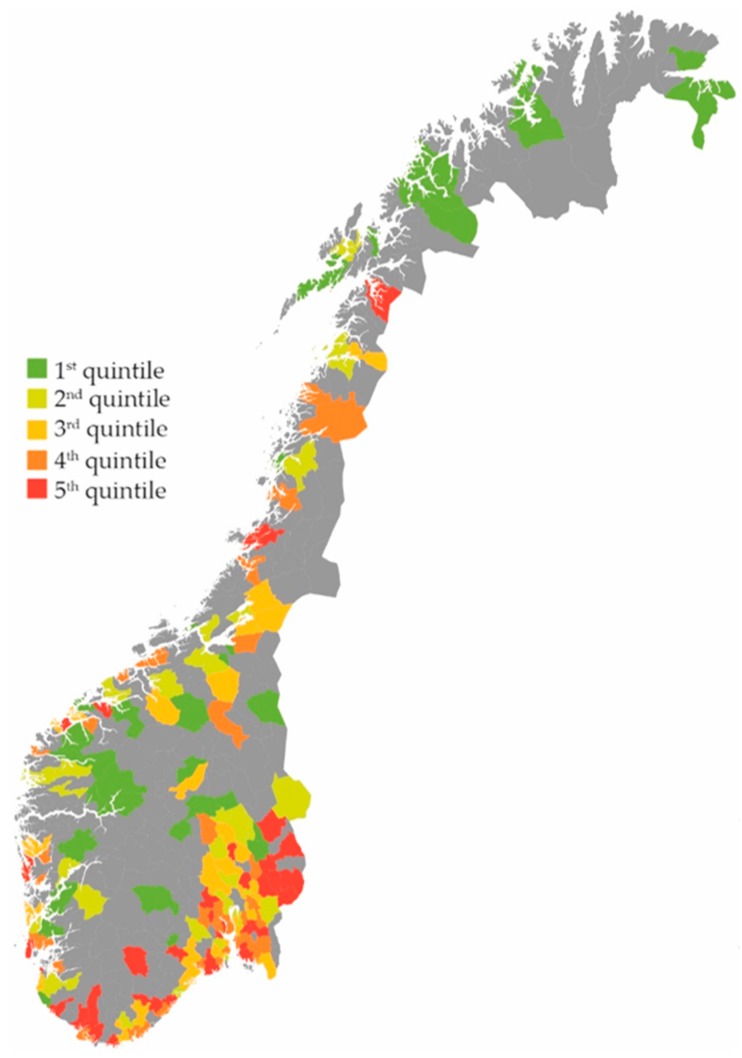
RTI antibiotic prescriptions per 1000 inhabitants in 198 Norwegian municipalities. Quintile 1: 76–186 prescriptions. Quintile 2: 186–209 prescriptions. Quintile 3: 209–224 prescriptions. Quintile 4: 224–246 prescriptions. Quintile 5: 246–331 prescriptions [13].

**Figure 2:**
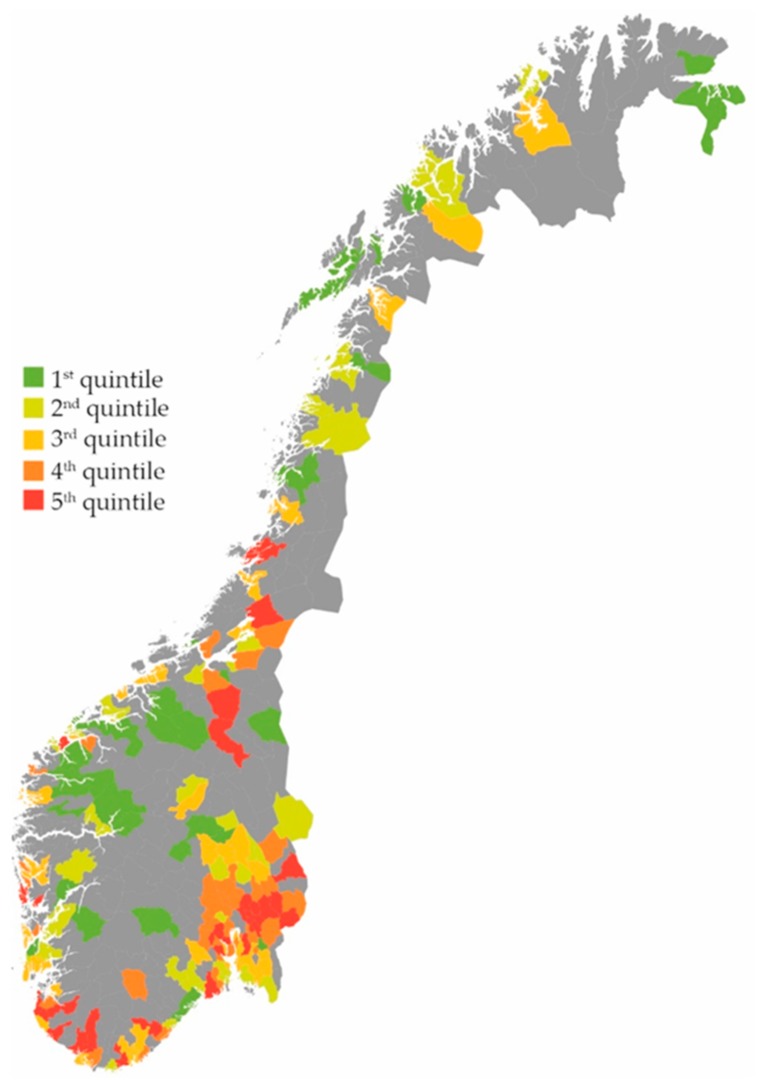
RTI consultations per 1000 inhabitants in 198 Norwegian municipalities. Quintile 1: 158–263 consultations. Quintile 2: 266–293 consultations. Quintile 3: 294–314 consultations. Quintile 4: 315–344 consultations. Quintile 5: 344–412 consultations [13].

**Figure 3:**
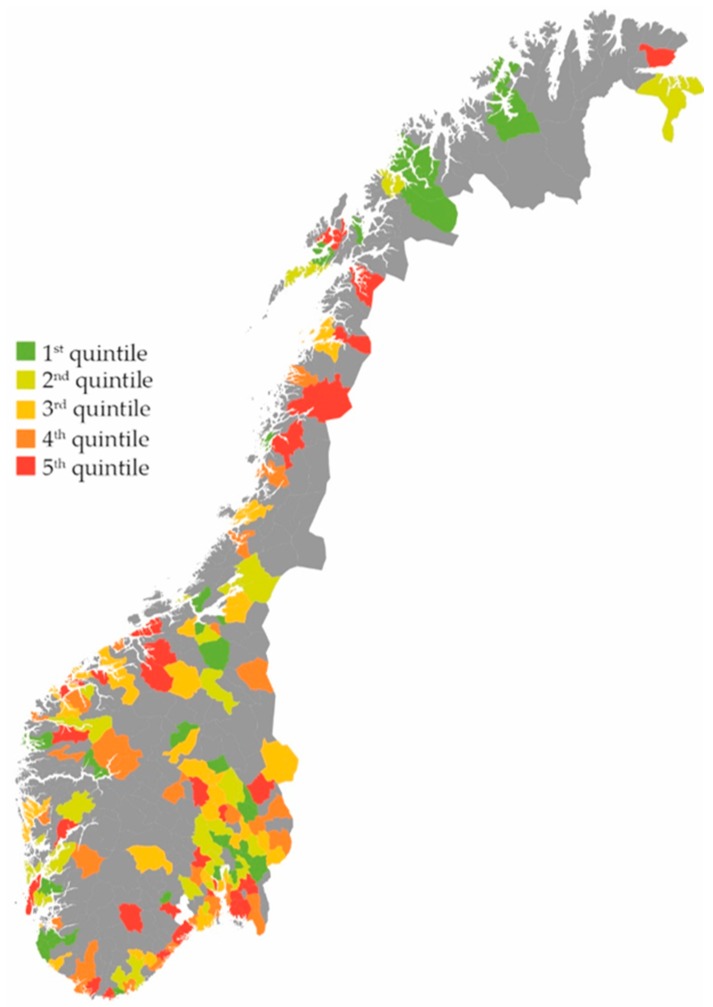
RTI antibiotic prescriptions per 1000 RTI consultations in 198 Norwegian municipalities. Quintile 1: 271–634 prescriptions per 1000 RTI consultations. Quintile 2: 636–688 prescriptions per 1000 RTI consultations. Quintile 3: 688–736 prescriptions per 1000 RTI consultations. Quintile 4: 738–792 prescriptions per 1000 RTI consultations. Quintile 5: 793–1146 prescriptions per 1000 RTI consultations [13].

Inhabitants with RTIs received 42% more prescriptions if they belonged to a municipality with high consultation rates versus low consultation rates (5th versus 1st quintile of consultation rates, (IRR) 1.42 (95% CI 1.41–1.44)). Furthermore, inhabitants with RTIs received 48% more prescriptions if they belonged to a municipality with high prescription rates versus low prescription rates (5th versus 1st quintile of prescription rates, IRR 1.48 (95% CI 1.47–1.50). Total numbers per municipality on RTI prescriptions, RTI consultations and RTI prescriptions per consultation are demonstrated in Figure 1, Figure 2 and Figure 3.

## 3. Discussion

### 3.1. Main Findings

Our study demonstrates that at a municipality level, inhabitants’ consultation rates and GPs’ prescription rates were almost equally associated with the number of RTI antibiotics prescribed.

### 3.2. Strengths

A municipality with less than 5000 inhabitants may be served by only a few GPs in Norway. By excluding such municipalities our aim was to reduce bias from doctors with markedly different prescription behaviour. Data were collected from national registries and covered more than 66% of the Norwegian population. In NorPD, the number of registered prescriptions is linked to the dispensing of a prescription from a pharmacy, thereby avoiding bias from delayed prescriptions that are not dispensed. In KUHR, the number and types of consultations that are registered are linked to reimbursements to health care providers. The financial incentives by reporting is high and we therefore assume that the numbers form KUHR are realistic.

### 3.3. Limitations

This study has some limitations. Data on consultations are collected from primary care (GP offices, A&Es), while data on prescriptions include all prescriptions dispensed outside institutions, including prescriptions from outpatient clinics or from specialist practices. A Danish study has shown that GPs prescribe about 75% of all antibiotic prescriptions dispensed at pharmacies [14]. Although GPs prescribe most antibiotics, our study does not adjust for geographic variation in density of specialists, which is therefore an unmeasured confounder in the study. The indication or diagnosis leading to a prescription is not registered in NorPD. We have linked RTI antibiotics with RTI consultations based on predefined ICPC-2 codes and ATC codes. This limiting of antibiotics included by use of predefined codes may have introduced bias. The method is vulnerable because antibiotics commonly indicated for RTIs have other indications, for example skin infections and sexually transmittable infections. We have not included telephone consultations or reiterations of prescriptions. Due to these factors, the calculated antibiotic prescription rate in our study is considerably higher than what is found when exploring electronic patient records from Norwegian general practice [15]. Our data are grouped on a municipality level. The results of the study therefore provide limited insight to the behaviour of individual GPs or patients. Furthermore, we did not include patient comorbidity as a confounding variable.

Moreover, this study does not address factors that affect health seeking behaviour or GP prescription behaviour in a municipality. Such factors are demonstrated to include the travel distance to the health care provider, socio-economic status, cultural differences in disease coping strategies and attitudes towards using antibiotics among inhabitants, as well as characteristics of the GP practice [16,17,18]. Studies have also demonstrated the association between overuse of antibiotics and increased patient re-attendance, leading to more prescriptions of antibiotics [19]. In our model, the variables are adjusted, so that the IRR is the mutual importance of prescription rates and consultation rates. Our model does not, however, identify which patients are frequently consulting primary care and which doctors are high prescribers. We have merely studied the effect of consultation rates and GPs’ prescription rates in themselves, regardless of factors that explain them. Despite these limitations we believe the results are useful and realistic when comparing to what degree consultation rates and prescription rates contribute to differences in antibiotic consumption in different municipalities.

### 3.4. Comparison with Existing Literature

Previous Norwegian studies report that RTI consultations comprise 11.7–15.0% of all consultations in primary care [15,20]. Our study finds lower consultation rates for RTIs with a share of 10.3% of all consultations. Furthermore, the share of RTI prescriptions has been reported as 51.3–57.7% of all dispensed antibiotic prescriptions in Norway [21,22]. This is in line with our findings of RTI prescriptions comprising 49.9% of all dispensed antibiotic prescriptions.

From 1994 to 2000 the number of antibiotic prescriptions for RTIs in British primary care was almost cut by half. In the same period, doctors had become more restrictive prescribers but a more important explanation to the reduction was that the population less often sought medical attention for RTIs [11,12]. Our results are in line with this; not only chronological variations but also geographical variations, can to a large extent be explained by variations in health seeking behaviour.

A recently published study has investigated the characteristics of patients consulting their GPs for suspected RTIs. These same characteristics did not seem to affect the doctor’s subsequent decision to prescribe antibiotics [23]. Patients report that the most important reasons to attend their GP include symptom relief and assurance, not necessarily an antibiotic prescription [24]. These findings stress that patients attending their GP with a suspected RTI are not necessarily the patients with most benefit of antibiotics.

It has been said that the most important risk factor for receiving an unnecessary antibiotic prescription is consulting a doctor [25]. In our opinion, patients’ health seeking behaviours have been underappreciated, both as an explanation and a factor affecting the large number of unnecessary antibiotic prescriptions made in primary care.

## 4. Materials and Methods

### 4.1. Data Collection

The number of antibiotic prescriptions were collected from The Norwegian Prescription Database (NorPD) [26]. NorPD at the Norwegian Institute of Public Health monitors all drugs dispensed by prescription outside institutions (hospitals, nursing homes) in Norway. The number of primary care consultations were collected from the Directorate of Health’s system for control and payment of health reimbursements (KUHR) registry [27]. The KUHR registry is a national database collecting data on the number and types of consultations as a part of the reimbursement system for health care providers. Population statistics including age and gender were collected from Statistics Norway (SSB) [28]. All data were on a municipality level and from 2014.

### 4.2. Inclusion and Exclusion Criteria

A total of 428 municipalities existed in Norway in 2014 [29], with a median number of inhabitants of 4600. In small municipalities, there may be long travel distances to pharmacies and GP offices and A&Es may provide patients with antibiotics directly. Such consumptions are not registered in NorPD. To ensure data validity and avoid missing numbers, only municipalities with 5000 inhabitants or more in 2014 were included (*n* = 202). The four largest municipalities (Oslo, Bergen, Trondheim, Stavanger) were excluded from the study because we expected a higher share of private health care providers from which data on consultations are not registered in KUHR. A total of 198 municipalities were included in the study.

### 4.3. Variable Definition

Age was categorised as early childhood (0–9 years), late childhood and adolescence (10–19 years), young adults (20–29 years), adult and early retirement age (30–79 years) and late retirement age (>80 years). A primary care consultation was defined as either an appointment at a GP office, a home visit or a consultation at an A&E unit. A consultation for an RTI was defined as a consultation registered with one of the following International Classification of Primary Care 2 (ICPC-2) codes: R01–05, R07–29, R71, R72, R74, R75, R76, R77, R78, R80, R81, R82, R83, H01, H71, H72, H74 (Table 4) [30]. This is the same definition as has been used in earlier research on RTIs in Norwegian ambulatory care [31]. Antibiotics with RTIs as the presumed most frequent indication were defined as an RTI antibiotic (Table 5). Inhabitants’ consultation rates for RTIs were defined as the number of RTI consultations per 1000 inhabitants per year. GPs’ prescription rates for RTIs were defined as the number of antibiotic prescriptions for treatment of RTI per 1000 RTI consultations per year.

### 4.4. Missing Data

SSB contained no missing data. In NorPD, to ensure anonymity of inhabitants in a municipality, data was reported as missing if the number of prescriptions were less than 5 for a given age and gender group. This resulted in 0.15% of data reported as missing for RTI prescriptions. 6.11% of data were missing for RTI consultations.

### 4.5. Modelling

We have used a mixed Poisson regression model to evaluate the effect of age, gender, GPs’ prescription rates and inhabitants’ consultation rates on the outcome of interest, which was RTI antibiotic prescriptions per 1000 inhabitants. Different age groups show varying consumption rates of antibiotics (Table 1). To account for this, inhabitants’ consultation rates and GPs’ prescription rates were categorised by age and gender adjusted quintiles before being included in the model. Quintiles were chosen as fewer groups would provide less accuracy and additional groups would make the calculations extensive. A mixed model was used to account for the clustering of data on a municipality level. The two software packages used for analysis were Microsoft Excel for Windows version 14, IBM SPSS Statistics for Windows version 22.0 and STATA/SE version 14.1 for Windows.

### 4.6. Ethics

Data was collected from national registries. The raw data set contained the total number of persons, prescriptions and consultations for each gender and age group per municipality. The data was not linked to personal identification numbers. Furthermore, if the number of consultations or prescriptions for a given age and gender group was less than 5, the registry described this number as anonymous. As all data were anonymous and aggregated, no study approval was applied for.

## 5. Conclusions

Inhabitants’ consultation rates and GPs’ prescription rates have an almost equally strong association with differences in antibiotic consumption between Norwegian municipalities. Our study highlights the importance of targeting both patients and doctors in efforts to reduce unnecessary antibiotic prescriptions in primary care.

## Figures and Tables

**Table 1:** Number of included females and males by age group.

Age	Number of Included Females	Number of Included Males	Total
0–9 years	201,493	212,574	414,067
10–19 years	212,673	224,929	437,602
20–29 years	201,200	214,863	416,063
30–79 years	968,954	983,760	1,952,713
80 years or older	90,299	53,843	144,142
Total	1,674,618	1,689,968	3,364,585

**Table 2:** Respiratory tract infections (RTI) consultations per 1000 inhabitants, RTI antibiotic prescription per 1000 RTI consultations and RTI antibiotic prescriptions per 1000 inhabitants in 198 Norwegian municipalities in Norway in 2014.

Age	Gender	RTI Consultations per 1000 Inhabitants (SD)	RTI Antibiotic Prescriptions per 1000 RTI Consultations (SD)	RTI Antibiotic Prescriptions per 1000 Inhabitants (SD)
0–9 years	Female	604 (129)	356 (67)	217 (66)
	Male	672 (139)	345 (67)	234 (73)
10–19 years	Female	295 (56)	570 (107)	166 (40)
	Male	206 (46)	552 (134)	110 (27)
20–29 years	Female	352 (58)	797 (123)	278 (52)
	Male	209 (43)	809 (177)	165 (35)
30–79 years	Female	307 (44)	853 (136)	260 (48)
	Male	211 (29)	940 (146)	197 (36)
80 years or older	Female	282 (66)	896 (238)	243 (66)
	Male	353 (100)	972 (293)	325 (94)
	Total ^1^	311 (145)	779 (236)	220 (62)

^1^ Weighted mean.

**Table 3:** The effect on total use of antibiotics for RTIs, mixed linear Poisson regression model.

Variable	Antibiotic Prescriptions for Treatment of RTI		
	Incidence ^1^	IRR	95% CI
**GPs’ prescription rates**			
1st quintile	187	1	
2nd quintile	201	1.11	1.10–1.12
3rd quintile	216	1.20	1.19–1.22
4th quintile	233	1.32	1.30–1.33
5th quintile	258	1.48	1.47–1.50
**Inhabitants’ consultation rates**			
1st quintile	184	1	
2nd quintile	208	1.11	1.10–1.12
3rd quintile	225	1.18	1.17–1.20
4th quintile	227	1.29	1.27–1.30
5th quintile	246	1.42	1.41–1.44
**Age**			
0–9 years	229	1	
10–19 years	137	0.62	0.61–0.63
20–29 years	218	0.99	0.98–1.00
30–79 years	232	1.01	1.01–1.02
≥80 years	277	1.18	1.16–1.19
**Gender**			
Female	247	1	
Male ^2^	192	0.78	0.78

^1^ Weighted incidence: antibiotic prescriptions per 1000 inhabitants per year. ^2^ Additional decimal values: Male IRR = 0.7808, 95% CI = 0.776–0.784. GPs’ prescription rates: municipality-level antibiotic prescriptions per 1000 consultations per year. Inhabitants’ consultation rates: municipality-level consultations per 1000 inhabitants per year. IRR is adjusted for age and gender. All *p*-values < 0.05.

**Table 4:** International Classification of Primary Care 2 (ICPC-2) codes and corresponding descriptions.

ICPC-2 Code	Description
R01	Pain respiratory system
R02	Shortness of breath/dyspnoea
R03	Wheezing
R04	Breathing problem, other
R05	Cough
R07	Sneezing/nasal congestion
R08	Nose symptom/complaint other
R09	Sinus symptom/complaint
R21	Throat symptom/complaint
R23	Voice symptom/complaint
R24	Haemoptysis
R25	Sputum/phlegm abnormal
R26	Fear of cancer respiratory system
R27	Fear of respiratory disease, other
R28	Limited function/disability (r)
R29	Respiratory symptom/complaint, other
R71	Whooping cough
R72	Strep throat
R74	Upper respiratory infection acute
R75	Sinusitis acute/chronic
R76	Tonsillitis acute
R77	Laryngitis/tracheitis acute
R78	Acute bronchitis/bronchiolitis
R80	Influenza
R81	Pneumonia
R82	Pleurisy/pleural effusion
R83	Respiratory infection other
H01	Ear pain/earache
H71	Acute otitis media/myringitis
H72	Serous otitis media
H74	Chronic otitis media

**Table 5:** Classification of antibiotic prescriptions by Anatomical Therapeutic Chemical (ATC)-codes.

ICPC-2 Code	Description
J01AA02	Doxycycline
J01CA04	Amoxicillin
J01CE02	Phenoxymethylpenicillin
J01FA	Macrolides
